# The Active with OsteoArthritis (AktivA) Physiotherapy Implementation Model: A Patient Education, Supervised Exercise and Self-Management Program for Patients with Mild to Moderate Osteoarthritis of the Knee or Hip Joint. A National Register Study with a Two-Year Follow-Up

**DOI:** 10.3390/jcm9103112

**Published:** 2020-09-26

**Authors:** Inger Holm, Are Hugo Pripp, May Arna Risberg

**Affiliations:** 1 Department of Interdisciplinary Health Sciences, Oslo Norway/University of Oslo, Medical Faculty, 0318 Oslo, Norway; 2 Division of Orthopaedic Surgery, Oslo University Hospital, 0427 Oslo, Norway; m.a.risberg@nih.no; 3 Oslo Centre of Biostatistics and Epidemiology, Research Support Services, Oslo University Hospital, 0427 Oslo, Norway; apripp@ous-hf.no; 4 Department of Sports Medicine, Norwegian School of Sport Science, 0863 Oslo, Norway

**Keywords:** physiotherapy, implementation of guidelines, osteoarthritis, patient education, exercise therapy, physical activity, patient-reported outcomes

## Abstract

Background: Recent systematic reviews and international guidelines recommend patient education, exercises, and weight control (if overweight) as first-line treatment for patients with hip or knee osteoarthritis (OA). The Active with osteoArthritis (AktivA) program is a physiotherapy model for the implementation of these guidelines into clinical primary care practice. The purpose of the present study was to evaluate the long-term effects of and adherence to the AktivA program for patients with mild to moderate knee or hip OA. Methods: The AktivA program includes three modules: a physiotherapy certification course, a patient education and exercise program and an electronic quality register. An electronic questionnaire including questions about, pain, quality of life, physical activity, self-efficacy and satisfaction with the AktivA program are sent to the participants at inclusion and after 3, 12 and 24 months. A linear mixed model for repeated measurements was used to assess the difference between the follow-up times. Results: Until January 2020, 6245 patients were included in the register. The response rates were 98%, 86% and 63% at 3, 12 and 24 months, respectively. After participating in the AktivA program, the patients reported decreased pain and increased health-related and disease-specific quality of life at three months and the positive effect was maintained up to two years after inclusion. The proportion of patients reporting to be inactive or having a low physical activity level was reduced from 43% to 22%. After two years, more than 80% of the participants reported to use what they have learned from the AktivA program at least once a week. Conclusions: Two years after inclusion in the AktivA physiotherapy program, the patients still report reduced pain, increased quality of life and higher activity levels.

## 1. Introduction

Clinical guidelines for knee and hip osteoarthritis (OA) are based on high-quality randomized controlled trials, systematic reviews [1,2,3] and consensus agreements from international expert groups [4,5]. Kraus et al. [6] recently published a systematic umbrella review to evaluate the impact of physical activity on knee and hip osteoarthritis (OA). They concluded, yet again, that physical activity, irrespective of mode, decreases pain, improves physical function and quality of life. A physically active lifestyle provides significant arthritis-related benefits and it is highly unlikely that these conclusions will be changed by initiating new RCTs. Hunter and Bierma-Zeinstra provided an update on osteoarthritis in Lancet, where the message was that self-management and education, exercise (including both strengthening and aerobic exercise) and weight control, should be recommended as a first-line treatment for all patients with OA [7]. 

Despite a high volume of evidence for “what works” for patients with OA, clinical guidelines are inadequately applied in practice by health professionals [8,9], inadequately integrated into lifestyle behaviors by health consumers [10,11], and inadequately addressed by health policy and health delivery services in relation to the huge burden of the disease [12,13]. The intention of clinical guidelines is to implement evidence-based treatment approaches and to ensure that all patients with a given health problem or diagnosis are offered the best suitable and effective treatment alternatives available at any time [14]. However, BMJ Open recently published a systematic review where the aim was to determine what percentage of physical therapy treatment choices for musculoskeletal conditions agreed with management recommendations in existing guidelines [15]. The authors found that many physiotherapists seem not to follow evidence-based guidelines. They encourage physiotherapists to increase the frequency with which they provide evidence-based treatment choices and to reduce the use of undocumented modalities [15]. The implementation of new guidelines is essential to change existing practice and improve treatment quality, but the translation of such treatment recommendations into routine practice is a complicated process with many barriers, such as an insufficient knowledge of current guidelines, and finding it hard to break old habits and too complicated to employ new routines [14].

Over the last decade, several OA implementation models and OA programs for use in clinical practice have been introduced. In Sweden, the nationwide program “Better management of patients with osteoarthritis” (BOA) [16], was initiated 2008. In 2013, the Danish model “Good Life with osteoarthritis in Denmark” (GLA;D) [17] was launched. Both models include three main parts: 1. a certification course for health care professionals, 2. a patient education and supervised exercise program and 3. a nationwide quality register. Both programs have shown promising results—participation decreases pain, increases quality of life and self-efficacy [16] and has a significant impact on the intake of painkillers and sick leave up to 1 year after inclusion [17,18]. In 2016, a similar implementation model, the AktivA (Active with osteoArthritis), was initiated in Norway. Patient reported data were collected over a two-year period, which enables the evaluation of long-term adherence with and effects of the program.

For many OA patients, the international guidelines imply a change of behavior by introducing both systematic physical activity habits and a healthy diet and on permanent basis. It is a demanding and time-consuming process and individually tailored OA education and self-management strategies might be necessary to give the patients the tools they need to continue their “new” lifestyle in a lifelong perspective. Individual decision-making is an important focus to engender intentional change [19].

The main purpose of the present study was to evaluate the long-term effects on pain, quality of life, physical activity, self-efficacy and adherence to the AktivA program for patients with mild to moderate knee or hip osteoarthritis.

## 2. Materials and Methods

The present study is based on data retrieved from the AktivA quality register. Patients gave their electronic or written consent to be included in the register. A license to collect and process personal data was given from The Norwegian Data Protection Authority Inspectorate. Clinical trial registration was not necessary. The Regional Committee for medical and health research ethics gave their approval to analyze data and publish results from the register (2018/1572/REK).

### 2.1. Subjects

Patients may be referred from general practitioners, orthopedic surgeons or from making contact with a physiotherapist on their own initiative. The inclusion criteria were: age ≥ 35 years and clinical symptoms compatible with hip or knee OA or early signs of knee OA such as degenerative meniscal tears. The diagnosis only had to be verified by a clinical examination based on clinical criteria alone, using the EULAR evidence-based recommendations for the diagnosis of mild/moderate OA: pain stiffness and functional limitations [20]. Radiographic verifications were not necessary, as the restricted use of radiological examinations is strongly recommended.

### 2.2. The AktivA Program

AktivA is a modular program, and includes several components, which have been designed to follow international guidelines and provide optimal treatments for OA patients. The program includes three modules: a physiotherapy certification course, a patient education and exercise program and an electronic quality register.

### 2.3. The Physiotherapist Certification Course

The physiotherapist certification course is a one-day course, including theoretical knowledge about OA, updated evidence on first-line OA treatment, addressing dose-response principles for exercise programs, physical activity strategies and lifestyle changes. It also includes information about the AktivA program (organization, inclusion/exclusion criteria, extent of the program/minimal intervention, etc.), and practical training including the use of the electronic registration system and exercise demonstration. The lecturers are a multidisciplinary group including physiotherapists, an orthopedic surgeon, user representatives, a nutritionist and a specialist addressing communication skills. Two weeks before attendance, the participants are given access to the digital education tools (Power Point presentations and instructional videos) and the quality register. At the end of the course, the participants received an approval as a certified AktivA physiotherapist.

### 2.4. The Patient Education and Exercise Program

The patient education program includes an osteoarthritis school (class instruction for 3 h) and an individually tailored and supervised exercise program (lasting 6–12 weeks) provided by an AktivA physiotherapist. The osteoarthritis school is a structured program where patients receive information about OA (symptoms and signs, risk factor, weight control, treatment, etc.), the importance of physical activity and exercise, self-management skills (giving the patients the ability to strengthen their knowledge and actions to change their behavior concerning physical activity and exercise) and communication with peers living with OA. User representatives play an important role during both the osteoarthritis school and the exercise program, sharing their experiences with their “new mates”. The exercise therapy program is based on international guidelines and a previously published exercise program [2,21,22]. The AktivA program includes a set of 15 exercises intended to improve muscular strength, balance and functional stability (knee flexion and extension, squats, step-ups, leg press, balance, etc.). The number of exercises, detailed instructions in performing the exercises chosen correctly, dose and progression are individually delivered to each patient, based on their physical activity level and experience with exercise training. In communication with the physiotherapist, the patients identify skills and/or activities they want to improve. Individual goal setting is highlighted. When driven by the patient’s own decisions, the chance of success more realistic. Instructional videos showing the performance of each exercise are available for both the patients and the physiotherapists on AktivA’s website (www.aktivmedartrose.no). The program should be performed at least twice a week (12–24 sessions during the supervised exercise period), either alone or together with other OA patients, under the supervision of an AktivA physiotherapist. The physiotherapist guides the patients with respect to detailed instructions on performing the exercises chosen correctly, dose and progression.

The exchange of personal experiences and user involvement is highly emphasized. The patients are encouraged to perform additional exercise sessions at the clinic and/or home exercises, to join already existing OA groups, where they can receive good advice and learn from patient experts. Such groups often create valuable unities, which helps the individual patients to initiate/continue their “new lifestyle” and to start or adjust their leisure time physical activities.

Both before entering the AktivA program and after three months, the patients meet for an individual visit with the physiotherapist. At the 3-month follow-up, the pain and function scores and PSFS scales are used to adjust the exercise program and treatment plan. After three months, the patients are expected to continue with the exercise and physical activity program on their own or together with peers. For exercise progression or for those in need of booster sessions or maintenance advice, patients are free to attend new visits with their physiotherapist.

### 2.5. The AktivA Quality Register

The electronic registration system (the Checkware software, www.checkware.com) includes carefully selected, internationally recommended and valid questionnaires of high methodological quality. To get access to the electronic registration system, the patients have to log in using their personal, electronic bank identification code (BankID). When first logged in, they receive an electronic consent form. If answering yes to participation in the AktivA register, the patient-reported outcome (PRO) assessments are sent at automatically scheduled times points (at inclusion and after 3, 12 and 24 months) through a link generated by the electronic system to the patient’s e-mail account. For patients not holding a bank identification code, the questionnaires, including a prestamped reply- envelop, are sent by mail. The electronic system generates summary reports, which allow immediate communication between the patient and the physiotherapist, making the results accessible as a summary report. The report can be used to adjust or change the patient’s treatment plan. In addition, the reports can be sent to the referring GP’s or orthopaedic surgeons. Data are stored at a secured server, and anonymous data are transferred to the research server at the university.

### 2.6. Outcome Measures

The register includes PROs about pain, disease-specific physical function, physical activity, quality of life, quality of care, disease burden, adherence and satisfaction with the AktivA program (Table 1).

Demographic data, such as age, gender, weight, height, education, living situation, and employment, were collected at baseline. Sick leave status was collected at all four time points.

Average hip/knee joint pain intensity during the last month is assessed on a Numeric rating scale (NRS) (0 = no pain, 10 = maximum pain).

For disease-specific questions about sports/recreational activities (SP) and quality of life (QoL), the Hip injury and Osteoarthritis Outcome Score (HOOS) [23] and the Knee injury and Osteoarthritis Outcome Score (KOOS) [24] were used. The two subscale scores range from 0 (worst) to 100 (best).

Health-related quality of life is assessed using the EuroQoL (EQ-5D-5L) tool. The EQ-5D global health (utility) index was calculated using the Danish tariff.

The patient-specific functional scale (PSFS) is used to identify important activities patients are unable to perform or are have difficulty with as a result of their OA problems [30]. The patients are asked to identify 1 to 3 functional activities they have difficulties to perform on an 11-point numeric rating scale from 0 (unable to perform the activity) to 10 (no problem performing the activity). PSFS was included for two reasons. First, it was used to identify the 1 to 3 most important functional activities the patients have personal difficulties with, for setting goals at baseline, second to adjust the exercise program and treatment plan and third, to evaluate changes in functional status over time.

Leisure time physical activity levels were calculated using the HUNT questionnaire [27]. The participants were asked about their exercise frequency (five categories from never to four or more days per week), intensity (no sweat, sweat or exhausted) and duration (less than 15, 15–30, 31–60 or more than 60 min). A HUNT index, to match with current recommendations to promote and maintain general health [31], was calculated [27]. The index includes four categories: “inactive” (no leisure time activities), “low” (activity level below recommended), “moderate” (activity level corresponding to current recommendations, either moderate intensity exercise for 150 min each week or 60 min of vigorous intensity, or a combination of these), and “high” (more active than the recommendation).

The revised Osteoarthritis quality indicator questionnaire (OA-QI) assesses patient-reported quality of care [28,32]. The items reflect current international treatment recommendations and were used to evaluate the quality of provided care prior to inclusion in the AktivA program.

Self-efficacy was evaluated using the Norwegian version of the Arthritis Self-efficacy Scale (ASES) and included the subscales for pain (five items) and symptoms (6 items). Each item is graded from 10 (very uncertain) to 100 (very certain) [29].

Adherence was registered both by the physiotherapists and by the patients. At the 3-month follow-up, the physiotherapists reported the number of sessions the patients had met for supervised exercise sessions in the clinic. In addition, the patients reported their exercise frequency as part of the HUNT questionnaire at baseline, 3, 12 and 24 months.

### 2.7. Statistical Analysis

Demographic data are presented as mean ± SD or frequencies in percent.

We used a linear mixed model for repeated measurements to assess the difference between the follow-up times. It was estimated with maximum likelihood with the follow-up time as a fixed effect and a subject-specific random intercept. The main outcome variables (pain, KOOS/HOOS Qol and SP) are graphically presented as trend plots using estimated marginal means with 95% confidence intervals. All regression analyses were conducted using Stata or SPSS statistics 26 statistical software program.

To assess whether there was a change in the proportion of patients on sick leave across the study period, Cochran’s Q test was used. Effect sizes (ESs) were calculated using the principle of Cohen’s *d* as the mean difference estimated from the linear mixed model divided by the SD at baseline), where ESs between 0.2 and 0.49, 0.5 and 0.8 and >0.8 were considered as small, medium and large, respectively [33].

## 3. Results

Between January 2016 and December 2019, 6245 patients gave their electronic consent and were included in the register. At 24 months, 1205 participants (19%) of the included patients had reached the two-year follow-up time point. The response rates were 98%, 86% and 63% at 3, 12 and 24 months, respectively.

A total of 3880 (62%) and 1863 (30%) patients considered the knee joint and hip joint as their most affected joint, respectively. A total of 502 patients considered other joints, such as hand or shoulder, as their most affected joint or did not respond to that question. Forty-four percent of the included patients were retirees, 40% were working full time or part time, and of them 17.5% were on sick leave due to OA. At inclusion, 49% of the patients reported that they had received information about OA, and 61% and 85% had received information about available treatment options and the importance of physical activity and exercise at previous contacts with health care personal. Sixty three percent, 25% and 39% of the patients reported to use painkillers such as paracetamol, opioids and NSAIDs, respectively. Seventy-five percent of the patients had undergone a radiographic examination, 49% of which had an MRI prior to inclusion in AktivA. Twenty-six percent had received a referral for a surgical evaluation (e.g., meniscal resection or joint replacement).

Ninety-four percent of the patients had participated in the AktivA osteoarthritis school (class session lasting for 3 h) and 78% completed 10–12 supervised exercise sessions.

Patient characteristics at inclusion are presented in Table 2. Except for bodyweight (BW), where the knee OA group included a significantly higher number of overweight and obese individuals than the hip OA group (76% versus 65%), there were no significant differences in baseline characteristics between the hip and the knee OA patients. For 48% of the patients, university was the highest educational level completed.

There was a significant change in the proportion of full-time or part-time working patients who were on sick leave due to OA across the two year period (*p* < 0.001); the proportion was reduced from 17.5% at baseline to 9.8% after two years.

**Figure 1:**
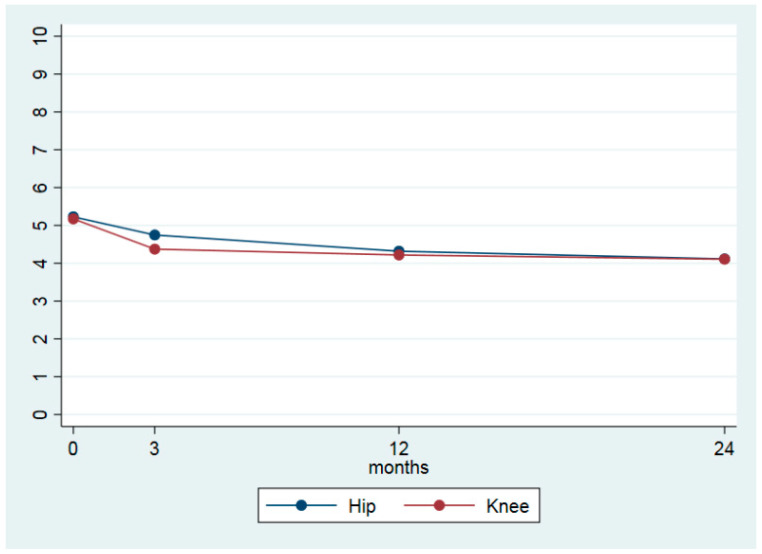
Pain during the last month (numeric rating scale 0–10) at baseline, 3, 12 and 24 months.

**Figure 2:**
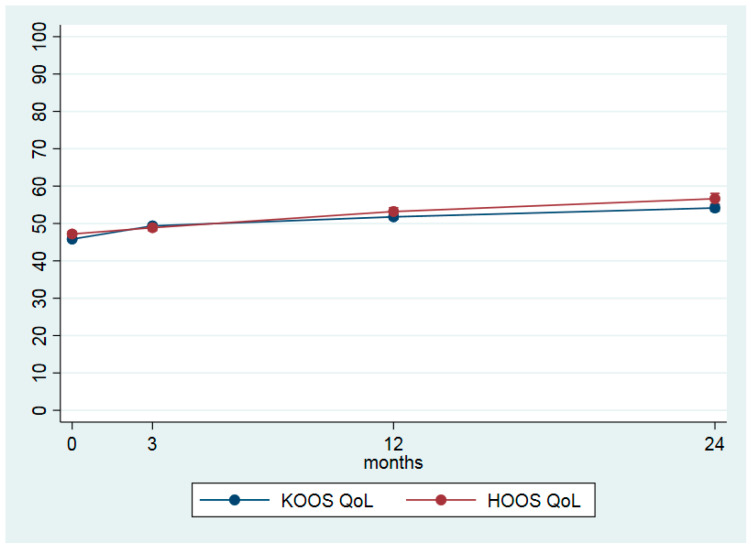
Joint related quality of life (QoL) (HOOS */KOOS **, scale 0 = worst–100 = best) at baseline, 3, 12 and 24 months given by estimated marginal means with 95% confidence intervals. * Knee injury and Osteoarthritis Outcome Score ** Hip disability and Osteoarthritis Outcome Score.

**Figure 3:**
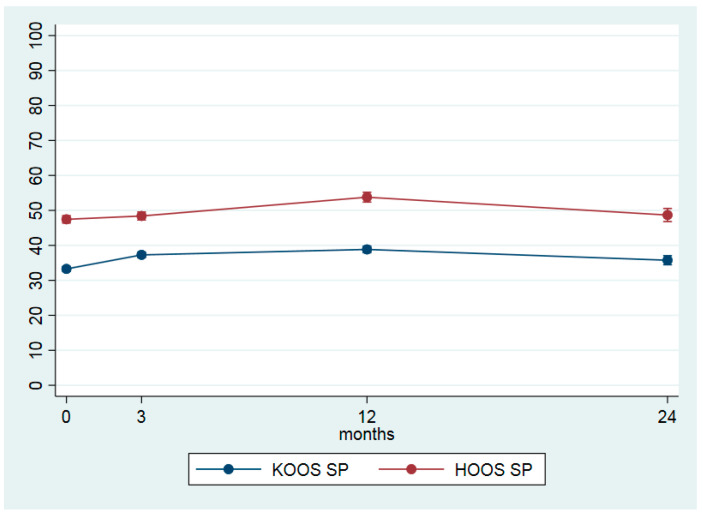
Difficulties in sport-related activities (SP) (HOOS */KOOS **, scale 0 = worst–100 = best) at baseline, 3, 12 and 24 months given by estimated marginal means with 95% confidence intervals. * Knee injury and Osteoarthritis Outcome Score ** Hip disability and Osteoarthritis Outcome Score.

Table 3 shows the differences between the four time points for the main outcome measures. From baseline to 24 months follow-up, the average pain in the last month was reduced from 5.2 (±1.8) to 4.1 (±2.2) points, giving an ES of 0.39 (Table 3 and Figure 1). For the 2-year period, KOOS QoL increased by 8.3 points and HOOS QoL by 9.4 points, resulting in an ES of 0.49 and 0.41, respectively (Table 3, Figure 2). Both values are within the recommended limits for a minimal clinical important change (http://www.koos.nu/koosfaq.html). For KOOS SP and HOOS SP only small changes were seen (Table 3, Figure 3). Health-related quality of life (EQ-5D VAS) increased from 64.1 points (±19.5) at baseline to 70.7 (±17.6) points at the 24-month follow-up. The EQ-5D utility index increased by 0.048, ES = 0.35 (Table 3). The calculated differences showed significant changes between all time points.

Thirty percent of the hip OA patients and 16% of the knee OA patients reported to have received a total joint replacement during the two-year follow-up period.

**Figure 4:**
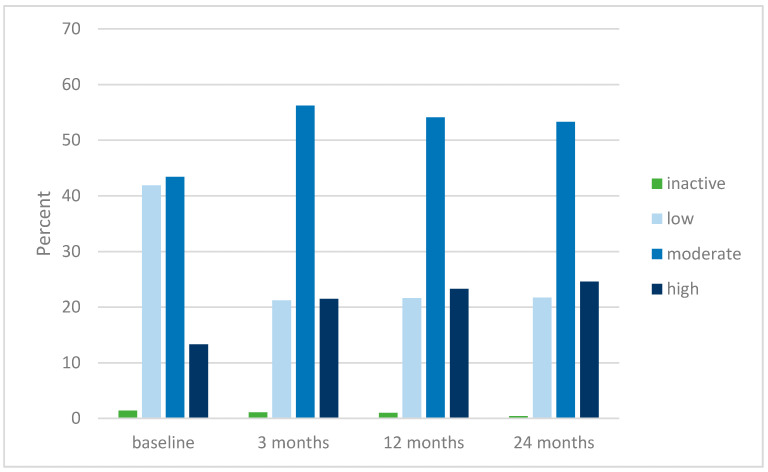
Distribution (given in %) of patient-reported physical activity levels at baseline, 3, 12 and 24 months in patients with knee or hip osteoarthritis who have participated in the Active with osteoArthritis (AktivA) education and supervised exercise program.

Figure 4 shows the distribution of patient-reported physical activity levels during the two-year follow-up. There was a significant change from baseline to three months, with the percent of patients reporting to be inactive or having a low activity level decreasing from 43% to 22%. The proportion of patients reporting inactivity or low activity level were 43%, 22%, 23% and 22%, at 0, 3, 12 and 24 months, respectively.

From inclusion to the 24 months follow-up, ASES pain and ASES symptoms showed significant changes, from 58.1 (±19.1) to 61.1 (±21.5) (*p* < 0.01) and 60.2 (±17.6) to 64.6 (±19.7) (*p* < 0.0001), respectively.

After two years, more than 80% of the participants still used what they have learned from the AktivA program at least once a week. Only 2.3% of the patients were unsatisfied with the program and dropped out.

## 4. Discussion

In a newly published editorial [34], Allen states that long-term outcomes of physical activity and exercise therapy programs for knee OA is relatively limited. The present study will at least add some new evidence to the field from an implementation study including several thousand patients. Three months after inclusion in the AktivA program, the patients reported decreased pain and increased health-related and disease-specific quality of life and the effects were maintained up to two years. The proportion of patients reporting to be inactive or having a low physical activity level was reduced from 43% to 22% and the results confirm that patients do not revert to prior lower physical activity patterns after the program ends.

OA affects a high number of adults, the risk of mobility disability is high [6] and the economic burden to society is substantial. It is therefore important to initiate evidence-based management strategies at an early disease stage, based on clinical signs and symptoms, even before radiographic signs occur. However, our results showed that 75% had undergone a radiological examination prior to inclusion and, of those, 49% had an additional MRI. Our results indicate that, in spite lack of indications for using MRI as a diagnostic method in routine clinical diagnosis [35], it is widely used even in patients with mild to moderate OA.

From baseline to the two-year follow-up, the NRS pain scores reduced from 5.2 to 4.1, which is defined as a minimal clinical important change [35], with an ES of 0.39 (Table 3 and Figure 1). These findings are in correspondence with the results both from the BOA [36], and GLA;D [17] registers, showing improvements of almost the same amount. The additional message from the present study is that the reduction in pain is maintained beyond the one-year period documented by the other registers. OA is a chronic condition with no cure, so one of the main treatment goals should be to reduce signs and symptoms moderately and to prevent worsening. The present results indicate that if we are able to teach individuals how to cope with their pain and symptoms and increase their physical activity level, they may experience a stable state of pain, without worsening, for years to come.

From baseline to two-year follow-up, KOOS and HOOS QoL showed a mean improvement of 8.3 and 9.4, respectively (Table 3 and Figure 2) which agree well with the findings from GLA;D [17]. The most interesting finding from the present study might be that the disease-specific QoL further improved from the one to two-year follow-up. The minimal important change (MIC) for KOOS is suggested to be 8–10 points [37] which indicates that the change in quality of life measured by KOOS should be considered clinically relevant to the patients. However, the MIC for KOOS is not calculated from a register-based population, so the results might be interpreted with some caution. The same positive tendency was seen for the general health related QoL (EQ-5D), where a small additional improvement was registered from one to two years. For KOOS and HOOS SP, only minor changes were seen during the study period (Table 3, and Figure 2), indicating that activities included in the questionnaire such as, e.g., running, jumping, squatting, kneeling, are improved. In the included OA patients, who had a mean age of 63.5 years, these activities might not have a high priority when it comes to resuming former activities.

At inclusion, more than 40% of the patients reported their physical activity to be low, and the proportion who did not engage in even small amounts of physical activity sessions was exactly the same as found in the recently published systematic umbrella review by Kraus et al. [5]. Our study confirms their findings and underlines the importance of initiating national implementation studies including an osteoarthritis school and supervised exercises to the inactive hip and knee OA patients as early as possible. The goal should be to make them confident living with their condition, teach them how to manage their daily life and to make them understand that an increased physical activity level will have beneficial impact both on their general health and OA condition, and will not harm them. Our results indicate that using such a strategy could result in a reduced number of sick leave references. However, this remains to be studied.

One specific goal during the AktivA education and exercise program is to provide the participants with simple tools for changing their physical activity behavior and engage in a more active lifestyle. It is important that they individually choose activities that they find pleasurable, and which they believe they could successfully integrate into their daily life routines and maintain on a permanent basis. At inclusion, 43% of the patients reported to be inactive or have a low activity level, at 3, 12 and 24 months the proportions of patients reporting to be inactive were reduced to 22%, 23% and 22%, respectively. The results indicate that the positive change in activity level achieved at 3 months was maintained up to the 24-month follow-up.

From baseline to the 3-month follow-up, we saw a marked shift in the distribution of activity level affiliation; the number of respondents reporting to be inactive/having a low activity level was reduced by almost 20%, the inactivity/low activity group was reduced from 43% to 22% and the change was maintained during the whole two-year follow-up period. Moderate amounts of physical activity, equivalent to 150 min per week of moderate intensity aerobic exercise, has been proven to be sufficient in reducing pain and not accelerate OA symptoms; therefore, our two-year results look promising with regard to the patients’ symptom control and physical fitness.

ASES evaluates one’s ability to undertake daily physical activities despite pain and other disease-related symptoms, and higher ASES scores are expected to correspond to lower levels of pain severity and impairment. The analysis from the present study showed significant, but clinically meaningful changes during the two-year study period, indicating that, despite decreased pain and increased quality of life (Table 3), the patients’ belief in their own ability to handle pain and other symptoms was almost unchanged. The results are in accordance with results from the BOA register [38], which showed almost identical self-efficacy levels at baseline and no changes after 12 months. In both studies, the mean baseline values for pain and symptoms ranged from 58 to 66, indicating that the patients are quite confident in their ability to master specific problems due to pain and other symptoms even at inclusion.

Using data from quality registers for the evaluation of treatment effects has some limitations. First, a register based on an electronic consent might be exposed to selection bias. The patients who want to be included in the AktivA program are persons who contact the health care system because of OA and who probably are interested in taking an active part in the treatment program. Patients who primarily wants medication and surgery might not want to be included in such a program and the present population is therefore not representative of the total population with mild/moderate OA. Second, in register studies including a high number of patients, unimportant differences might become statistically significant [39]. It is therefore important to interpret the results with caution and highlight results that are clinical important to the patients. Third, long-term follow-up registers including a high number of patients might, over time, be exposed to low response rates. In the present study, the response rates at the one- and two-year follow-up were 86% and 63%, respectively. The reason for the relatively high long-term response rates in the present study might be that the questionnaires are distributed in an electronic version. Our experience is that even older patients are online and master logging in to the system. Fourth, when selecting questions to be included in a quality register, it is important to limit the number of questions and follow the rule “need to know, not nice to know”. If the patients associate the questionnaire with a boring and time-consuming process, the response rate will rapidly drop. Fifth, the AktivA register only includes patient reported data, and so there are no objective measures of recovery. However, in the Nordic countries, all residents have their own personal identification number, which gives the opportunity to link different national registers together. The plan is to combine data from AktivA and the Norwegian Arthroplasty Register which, in the future, gives us the opportunity to combine patient-reported and objective measures, but for this purpose, we need a higher number of included patients.

The AktivA register does not include data to evaluate changes in PT knowledge, change of behavior, etc.—only self-reported data from the patients are included. We have a future intention of identifying facilitators and barriers for the implementation of the AktivA program in clinical practice. Our philosophy is that a pragmatic and simple approach for the implementation of new guidelines is crucial for achieving a permanent change [14].

## 5. Conclusions

Two years after inclusion into the AktivA program, the patients showed reduced pain, increased general and health-specific quality of life, and higher physical activity levels. More than 80% of the participants still used what they have learned from the AktivA program at least once a week. A crucial future challenge is to get more of the general practitioners and primary health care physiotherapists engaged in implementing the AktivA model and to include all hip and knee OA patients in the program at an early stage of disease.

## Figures and Tables

**Table 1:** Outcome measures included in the electronic registration at inclusion and 3-, 12- and 24-month follow-up.

	Baseline	3 Months	12 Months	24 Months
Demographic variables	x			
Height/weight	x	x	x	x
Pain (NRS) and complaints	x	x	x	x
HOOS/KOOS sports/recreational activities (SP) and disease-specific quality of life (QoL) [23,24]	x	x	x	x
Quality of life (EuroQol, EQ5D)	x	x	x	x
Patient-specific functional scale (PSFS) [25,26]	x	x	x	x
Physical activity (from HUNT questionnaire) [27]	x	x	x	x
Fear of physical activity	x	x	x	x
Osteoarthritis quality indicator questionnaire (OA-QI) [28]	x	x	x	x
Arthritis Self-efficacy scale (ASES) [29]	x	x	x	x

**Table 2:** Demographic data at inclusion for the total population and divided by the most affected joint.

	Total Population N = 6245	Knee OA N = 3880	Hip OA N = 1863
Age	63.5 ± 9.7	63 ± 9.7	64 ± 9.8
women/men, %	75/25	74/26	77/23
Body weight	82.7 ± 17.7	83.7 ± 16.6	78.5 ± 15.2
Body mass index	28 ± 4.9	28.6 ± 5	27 ± 4.6
Normal weight/overweight/obese, %	28/42/30	24/43/33	35/42/23
Pain intensity last month (Lickert scale, 0–10)	5.1 ± 1.9	5.2 ± 1.8	5.2 ± 1.8
Pain every day or always, %	81	80	81
On sick leave yes/no, %	17/83	18/82	16/84
ASES ^1^ pain	58 ± 19	60 ± 19	56 ± 19
ASES symptoms	60 ± 18	61± 17	59 ± 18
Physical activity level, low/moderate/high, %	57/12/31	56/13/31	56/12/32
Fear of physical activity, yes/no, %	20/80	22/78	16/84

^1^ ASES Arthritis self-efficacy scale, 10–100.

**Table 3:** Mean differences (SD) between baseline (T0) and 3 (T3), 12 (T12) and 24 (T24)-month follow-up, respectively.

Outcome	T0 Mean (SD ^1^)	T3 Mean (SD)	Mean Difference T0–T3 (CI ^2^)	Effect Size ^8^	T12 Mean (SD)	Mean Difference T0–T12 (CI)	Effect Size	T24 Mean (SD)	Mean Difference T0–T24 (CI)	Effect Size
Pain (NRS ^3^ 1–10)	5.2 (1.8)	4.5 (2.0)	−0.6 (−0.7–−0.6)	0.33	4.3 (2.1)	−0.8 (−0.9–−0.7)	0.37	4.1 (2.2)	−0.9 (−1.0–−0.8)	0.39
KOOS ^4^ sport (0–100)	33.3 (21.4)	37.4 (22.4)	4.0 (3.0–5.0)	0.21	39.2 (24.5)	5.6 (4.4–6.8)	0.26	36.3 (26.6)	2.5 (0.9–4.2)	0.11
KOOS QoL (0–100)	45.8 (14.5)	49.6 (15.4)	3.5 (2.9–4.2)	0.27	52.3 (17.0)	6.0 (5.1–6.8)	0.38	55.3 (18.3)	8.3 (7.2–9.5)	0.49
HOOS ^5^ sport (0–100)	47.5 (21.9)	48.7 (22.6)	1.0 (−0.5–2.4)	0.04	54.8 (25.2)	6.3 (4.5–8.1)	0.25	51.0 (26.5)	1.2 (−1.2–3.7)	0.02
HOOS QoL (0–100)	47.2 (14.8)	49.0 (16.0)	1.7 (0.6–2.8)	0.12	53.9 (17.7)	6.0 (4.6–7.4)	0.32	58.0 (19.9)	9.4 (7.5–11.4)	0.41
PSFS ^6^ 1 (0–10)	3.3 (2.4)	4.1 (2.9)	0.7 (0.6–0.8)	0.28	4.5 (3.1)	1.1 (0.9–1.2)	0.37	4.5 (3.1)	1.1 (1.0–1.3)	0.37
EQ-5D ^7^ health (0–100)	64.1 (19.5)	66.5 (19.5)	2.2 (1.5–3.0)	0.10	68.2 (18.7)	3.5 (2.5–4.4)	0.14	70.7 (17.6)	6.0 (4.7–7.3)	0.28
EQ-5D utility index (0–1)	0.721 (0.111)	0.746 (0.115)	0.025 (0.019–0.029)	0.16	0.759 (0.120)	0.036 (0.029–0.043)	0.27	0.773 (0.117)	0.048 (0.039–0.057)	0.35

^1^ SD, standard deviation, ^2^ CI, confidence interval, ^3^ NRS, numeric rating scale, ^4^ KOOS, Knee injury and osteoarthritis outcome score, ^5^ HOOS, Hip disability and osteoarthritis outcome score, ^6^ PSFS, Patient-specific Function Scale, ^7^ EuroQol 5D-5L, ^8^ Cohen’s *d* (mean difference/SD difference).

## Data Availability

Data will not be shared at this stage. Data from the present study are extracted as a part of a large, ongoing national register and several analyses are planned in the future.
